# Report of Committee on Diseases

**Published:** 1894-11

**Authors:** Leonard Pearson

**Affiliations:** Chairman; Phila.


### Report of Committee on Diseases, United States Veterinary Medical Association.

Phila., Sept., 1894.

Mr. President and Gentlemen :—Your Committee on Diseases has such an unlimited field that it is rather difficult to determine just what division of the subject should receive attention at this time. To attempt to catalogue all, or even all of the important, discoveries that have been made in the domain of comparative pathology and therapeutics during the past year, would necessitate the preparation of a paper altogether too long and too general for presentation at this time, and I fear that it would read too much like an abstract from the catalogue of a medical library.

If we attempt to make a selection from the published work of the past year, we shall call your attention to things that have already been introduced to you by the periodical medical and veterinary literature.

It seems well, therefore, to take a general survey of the subject of pathology, and, without going unnecessarily into the details of any of its branches, or delving into its past achievements, try to discover our present position as regards investigation of disease and its relation to the position of the veterinarian in other lands.

It is a lamentable fact that we are dependent, almost wholly, for our knowledge of comparative pathology upon the foreign schools. Up to the present time America has evolved but few original writers in this rich and promising field. Why is it that we are obliged to go abroad for an exact knowledge of the diseases that surround us at home? There is here no lack of material for study and no shortage of unexplored ground. Diseases abound that baffle our practitioners and confuse our students. Why is it that ambitious, energetic pathologists do not appear to grapple with the many obscure questions that make our daily work so difficult? Why, after all these years spent groping blindly in the mist, has no one told us more of puerperal apoplexy, anasarca, azoturea, cerebro-spinal meningitis, influenza, osteo porosis, and many other diseases, than our predecessors knew?

If this question were thoroughly sifted, it is likely that the answer would be found in the statement that "there is no money in it." In other words, the public has not been brought to see the importance of exact knowledge in regard to the intricacies of comparative pathology, and it has never been willing to pay for work that would lead to these ends.

There are, however, a few notable exceptions that prove the rule just stated. The National Government has established a most excellent Bureau of Animal Industry, which is officered in its research departments with thoroughly competent men, who deserve most of the credit that comes to this country for the advances it has made in the investigation of Texas fever, the contagious diseases of swine and some other diseases. The good work of this Bureau also in combatting and completely exterminating lung plague of cattle challenges the admiration of the world. While the Bureau of Animal Industry has many important discoveries to its credit, and has probably done as much good permanent research work in pathology as any other like number of scientists anywhere, the field is immeasurably too great for it to cover, and aid is needed from all quarters. There is no doubt there are many who would be glad to engage in research work if positions were opened to them and it were possible for them to exchange this work for a livelihood.

It is unnecesary to explain that the investigation of vague or unknown points in pathology is of the utmost value to the profession, and consequently to the public. Who then should encourage and support this work? There are but three factors, in addition to the Bureau of Animal Industry, to which we can look for assistance; the veterinary schools, the Agricultural Experiment Stations and veterinarians holding public positions. Of course private practitioners will make, and have made, many most important discoveries and advances, but these are chiefly in the realm of surgery and therapeautics. Advancements in pathology and bacteriology must come from the sources mentioned.

As yet, the schools have not done a great deal in this direction, from the fact that their teachers have been obliged to depend upon private practice for most of their income, the income of the schools not being sufficient to allow the payment of salaries large enough to command the entire energies of the professors. This difficulty is being overcome gradually in some of the endowed institutions, and it may be that, in time, the income of some of the private schools will be large enough to allow their teachers to devote more time to investigation. It should be understood that the function of the schools is not only to repeat and rearrange and dispense to students the knowledge that is at the disposal of anyone who wishes to search the libraries for it. They should seek to increase the existing stock of knowledge as well.

It is extremely important also that pathology should be well taught in the schools. It is the foundation of medicine, as anatomy is of surgery, and unless the students are well grounded in this branch, they will never be in a position to make valuable reports of cases or autopsies, or to intelligently interpret unusual conditions.

The Agricultural Experiment Stations should also support investigations in animal pathology. According to the provisions of of the Act of Congress under which they are established, it is expressly stated that "It shall be the duty of said Experiment Stations to conduct original researches or to verify experiments in the physiology of plants and animals and the diseases to which they are severally subject with remedies for the same." * * *

This provision has, in most cases, been ignored and the Experiment Stations that have conducted investigations in animal pathology are very few in number. The live stock interests are of sufficient magnitude and the diseases affecting our live stock are sufficiently numerous and disastrous to justify greater attention to this subject.

Why have the diseases of animals been so completely ignored or so parsimoniously treated by the Experiment Stations? Before attempting to answer this question, it should be stated that each station is at liberty to select the work that seems most important to the agriculturists and the public of the State in which it is located.

But why is it that some at least of the stations do not more fully recognize the importance of investigations of animal diseases? No other subject specifically mentioned in the act has received so little attention, notwithstanding the fact that this is the first on the list and was probably considered most important by the originator of this Experiment Station legislation. From some personal observations it has seemed to me that the reason is two-fold: 1st. The stations regard other work of more importance and do not wish to go to the expense of fitting out and supporting pathological and bacteriological laboratories, and 2nd, even when inclined to engage in this field the stations have difficulty in finding veterinarians accustomed to the work of investigation. Consequently, our schools should devote greater attention to the fundamental branches of medical science and endeavor to turn out men better fitted to grapple with new problems.

If one Experiment Station were to engage in this work on a sound basis, it is altogether probable that the good results achieved would lead other stations to follow the same course.

It is unfortunate that the abundant opportunities for original work that fall to the lot of some veterinarians holding public positions are not better utilized for the advancement of science.

If these opportunities for observation and investigation were better used, as they are, for instance, in Germany, it would tend to elevate the estimation in which the office-holding veterinarian is held. It would attract attention to the possibilities of his worth and usefulness and would have a tendency to open other positions wherein the veterinarian could be of great service to the public.

In this report of the Committee on Diseases, I have tried to call attention to the necessity for original work in pathology on the part of veterinarians in this country, and have pointed out the channels through which we must expect most of our advancement in the future.

As to the advances made in the past year, these are already known to you from other sources.

You have all remarked that much attention is being paid to blood serum therapy, and to the use of sterilized product of the growth of disease producing organisms in cultures in the detection or treatment of the diseases these organisms cause.

This field seems to be a very promising one, and many valuable results of great practical value have already been achieved by workers in it.

Much remains to be done before the antitoxins can be dispensed generally, as drugs are, but many think that this awaits us.

Contagious pleura pneumonia has not been discovered since the report at our Chicago meeting that the last case had been killed the year before So we have still further proof that this Association was justified in declaring this country free from the disease, and are now additionally sure that the United States is free from this plague.

Tuberculosis continues to attract much attention, and the relation of this disease to the State is rapidly becoming a prominent public question. Special questions will, however, be discussed in other papers.

LEONARD PEARSON, *Chairman.*

